# First person – Claire Montgomery and Lili Salinas

**DOI:** 10.1242/dmm.052364

**Published:** 2025-04-02

## Abstract

First Person is a series of interviews with the first authors of a selection of papers published in Disease Models & Mechanisms, helping researchers promote themselves alongside their papers. Claire Montgomery and Lili Salinas are co-first authors on ‘ Robust behavioral assessment of the inducible Friedreich's ataxia mouse does not show improvement with NRF2 induction’, published in DMM. Claire is a staff research associate in the lab of Drs Elena N. Dedkova and Gino Cortopassi at University of California, Davis, Davis, CA, USA, investigating mitochondria and their role in different metabolic diseases, drug development for rare mitochondrial diseases and mitochondrial toxicity. Lili is a PhD student in the same lab, investigating rare mitochondrial diseases, their pathophysiology and *in vivo* pharmacology in such diseases.


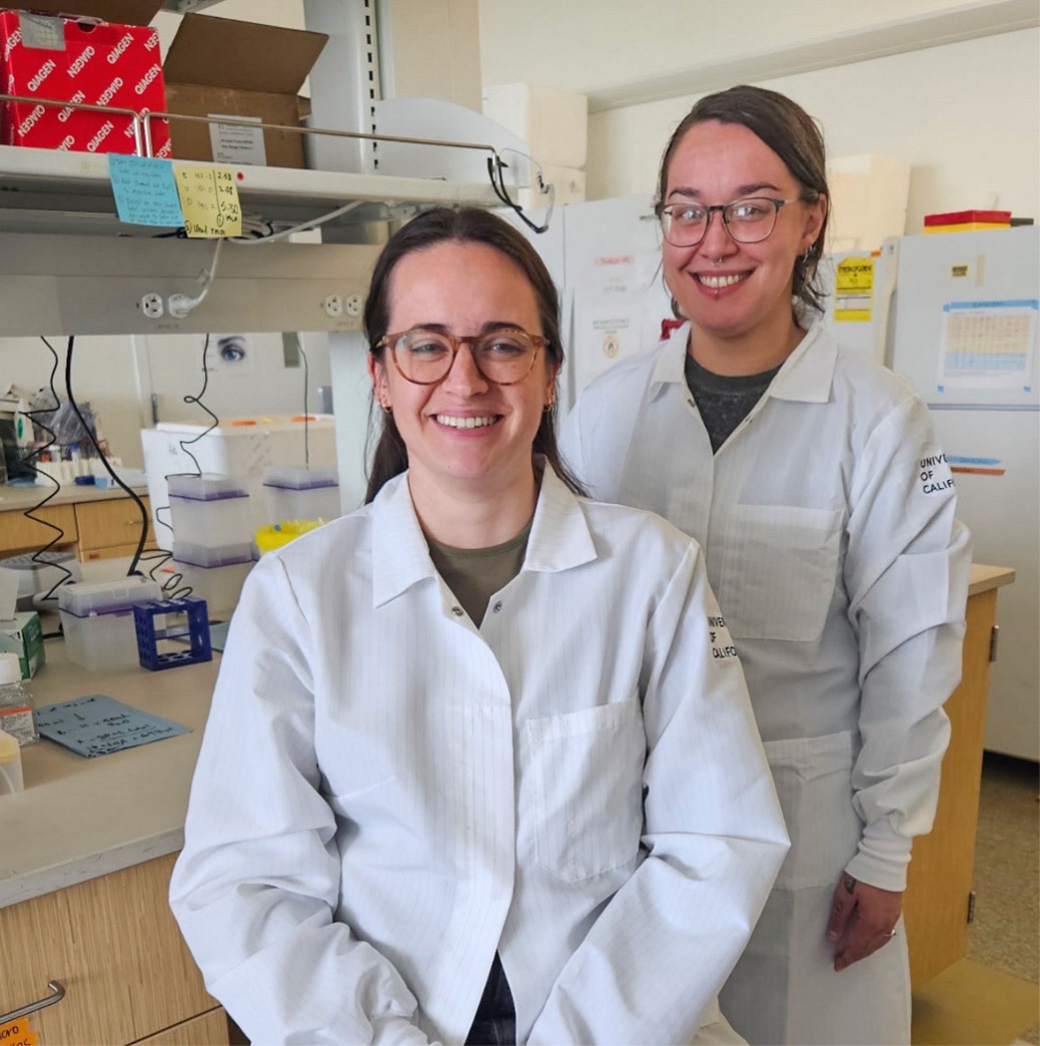


**Claire Montgomery (back) and Lili Salinas (front)**

**Who or what inspired you to become a scientist?**

**C.M.:** My mother is a very successful veterinarian who has always emphasized the importance of asking questions and following your hypothesis to the end. I was unsure what I wanted to do post-baccalaureate, and research was such a good fit during my senior year of my undergraduate degree that I fell in love with it. Over 6 years later, I am so glad I made that choice!

**L.S.:** I have watched both of my parents be diagnosed and cured from cancer during my life. This instilled in me a profound appreciation of science and a desire to help others through scientific discovery. Through some exploration, this had led me to the field of pharmacology, which has furthered my passion for scientific research.

**What is the main question or challenge in disease biology you are addressing in this paper? How did you go about investigating your question or challenge?**

Friedreich's ataxia (FA) is a rare neurological disease that leads to the impairment of movement coordination and walking abilities, and the development of scoliosis, diabetes and cardiomyopathy. Only recently, the first and only currently available drug (omaveloxolone) was approved for treatment of the neurological symptoms in FA. There are not too many mouse models of FA that can mimic both neurological and cardiac symptoms of disease, and the inducible mouse model of FA [also known as frataxin knockdown (FXNKD) model] mimics multiple symptoms of the disease. We developed a full battery of neurobehavioral assays, including novel Salinas–Montgomery ataxia scale (SMAS) score, to characterize this model in detail and test whether NRF2 agonists such as omaveloxolone and dimethyl fumarate could recover the neurological deficits observed in this model. Although this FA mouse model showed significant impairments in motor activity and neurobehavior, we did not find any significant beneficial effects of either omaveloxolone or dimethyl fumarate on neurological symptoms in this mouse model of FA.

[…] by adding several noninvasive tests, we were able to more precisely determine the severity of disease in these mice, and more accurately assess the effects of two drugs.

**How would you explain the main findings of your paper to non-scientific family and friends?**

FA is a rare genetic disorder that causes a progressive decline in motor function and heart health. This is caused by loss of a single protein found in the mitochondria (‘powerhouses of the cell’), known as frataxin. In this paper, we look at a specific FA mouse model in which we are able to test motor function decline and any improvements made by a drug intervention. We found that, by adding several noninvasive tests, we were able to more precisely determine the severity of disease in these mice, and more accurately assess the effects of two drugs.

**What are the potential implications of these results for disease biology and the possible impact on patients?**

We hope that these results cause the field to continue looking in new directions for treatments for FA, or combination therapies that tackle multiple facets of FA pathology. We believe that, although NRF2-inducing therapies may confer a small benefit in slowing behavioral decline in humans, more treatments need to be explored that also tackle the cardiomyopathy that causes shorted lifespan, as well as those that further prevent the neurobehavioral decline in both model organisms and humans.

**Figure:**
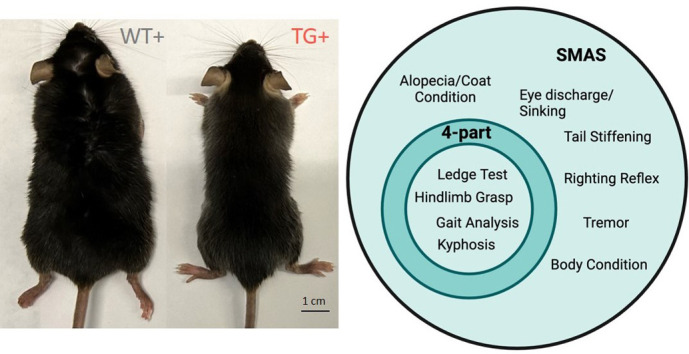
FXNKD mice show a severe ataxic phenotype, including signs of wasting and advanced aging (left). To better define this phenotype, we developed a novel observational test [the Salinas–Montgomery ataxia scale (SMAS)] that encompasses and surpasses the current standard, the four-part cerebellar ataxia scale (right).

**Why did you choose DMM for your paper?**

This paper further delves into the disease model of FA that appears to recapitulate both neurological and cardiac decline of the disease. In this work, we mostly focused on neurological and skeletal muscle deficits of this mouse model of FA. We felt that DMM would be a good home for our paper as this journal provides a platform to describe animal models of human disease.

**Given your current role, what challenges do you face and what changes could improve the professional lives of other scientists in this role?**

Currently, we are experiencing an interruption and some uncertainty in this country when it comes to scientific funding. This is affecting many people, including PhD students, as well as staff researchers and PIs at countless universities. The lack of belief in scientific research is causing feelings of instability in many careers. We believe that by focusing on problems that are facing humans right now, the field of research will continue to be supported in pursuit of treatments to help real humans that are currently suffering.

**What's next for you?**

**C.M.:** I am working on disseminating research in another one of my wheelhouses: environmental toxicology. Our group is also working on the toxicology behind quaternary ammonium compounds, which are known mitochondrial toxins.

**L.S.:** I am graduating from my PhD in June and currently trying to figure out what is next! I would like to continue to work on rare diseases and, ideally, mitochondrial diseases and mouse models of human disease.

**Tell us something interesting about yourself that wouldn't be on your CV**

**C.M.:** I almost got a second baccalaureate degree in music theory and ethnomusicology. I was 28 units away from achieving it, but prioritized my scientific career instead, and I’m sure glad I did!

**L.S.:** Outside of my professional research interests, I love learning about the use of psilocybin in psychiatric disorders. I enjoy reading about plant medicine as a complement to western medicine.
